# Four new species of *Ctenodrilus*, *Raphidrilus*, and *Raricirrus* (Cirratuliformia, Annelida) in Japanese waters, with notes on their phylogenetic position

**DOI:** 10.7717/peerj.13044

**Published:** 2022-03-08

**Authors:** Naoto Jimi, Shinta Fujimoto, Yoshihiro Fujiwara, Kohei Oguchi, Toru Miura

**Affiliations:** 1 Sugashima Marine Biological Laboratory, Graduate School of Science, Nagoya University, Toba, Japan; 2 Centre for Marine & Coastal Studies, Universiti Sains Malaysia, Penang, Malaysia; 3 Research Center for Marine Biology, Graduate School of Life Sciences, Tohoku University, Aomori, Japan; 4 Research Institute for Global Change (RIGC), Japan Agency for Marine-Earth Science and Technology (JAMSTEC), Yokosuka, Japan; 5 National Institute of Advanced Industrial Science and Technology, Tsukuba, Japan; 6 Misaki Marine Biological Station, School of Science, The University of Tokyo, Miura, Japan

**Keywords:** Annelida, Japan, New species, Pacific Ocean, Polychaeta, Taxonomy

## Abstract

Four new species of annelids, *Ctenodrilus japonicus* sp. nov., *Raphidrilus misakiensis* sp. nov., *Raphidrilus okinawaensis* sp. nov., and *Raricirrus anubis* sp. nov., are described based on specimens collected from Japanese waters. *Ctenodrilus japonicus* sp. nov. inhabits the interstitial environment and can be distinguished from the other congeners by the following features: (*i*) total of 16 chaetigers, (*ii*) chaetigers 1–3 with stout hooks, (*iii*) minute body (approximately 1 mm in length), (*iv*) all parapodia with the same number of chaetae (two notochaetae; two neurochaetae), and (*v*) presence of dorsal and ventral papillae. *Raphidrilus misakiensis* sp. nov. lives under intertidal stones and can be distinguished from other congeners by having pectinate neurochaetae. *Raphidrilus okinawaensis* sp. nov. inhabits the interstitial environment and can be distinguished from other congeners by: (*i*) absence of annulation on the peristomium and achaetous segment and (*ii*) presence of a heart body in chaetigers 4–5. *Raricirrus anubis* sp. nov. inhabits whale bones and can be distinguished from other congeners by the following features: (*i*) presence of a heart body in chaetigers 9–14, (*ii*) presence of capillary neurochaetae on chaetiger 1, and (*iii*) presence of simple curved spines. A phylogenetic tree based on partial sequences of cytochrome *c* oxidase subunit I and 16S rRNA from the new species and other cirratulid worms showed that *Raphidrilus* is included in Cirratuliformia. This is the first record of *Raphidrilus* and *Raricirrus* from Japanese waters.

## Introduction

Before the era of molecular phylogenetics, the following four genera characterized by their cirratuliform body of minute size and absence of dorsal tentacles, formed the family Ctenodrillidae Kennel, 1882: *Aphropharynx* Wilfert, 1974, known only from an aquarium of Aquazoo Löbbecke Museum Dusseldorf, Germany, with capillary chaetae, multidentate hooks, and lacking branchiae (Wilfert, 1974); *Ctenodrilus* Claparède, 1863 with multidentate hooks and body without branchiae, recorded from natural interstitial environments and aquaria (Magalhães et al., 2016); *Raphidrilus* Monticelli, 1910 with capillary chaetae and branchiae, recorded from thallus of algae or sand interstices (Magalhães, Bailey-Brock & Davenport, 2011); *Raricirrus* Hartman, 1961 with capillary chaetae, acicular spines, simple curved spines, and branchiae, recorded from chemosynthetic environments (sunk woods, whale bones, and thermal vent) (Petersen & George, 1991; Magalhães, Linse & Wiklund, 2017). Compared with other cirratuliform polychaetes, these genera favor specific environments such as chemosynthetic areas, where recent advances in sampling methods, namely ROVs, have revealed their biodiversity (Buzhinskaja & Smirnov, 2017; Magalhães, Linse & Wiklund, 2017). Although recent molecular phylogenetic analyses indicated that *Ctenodrilus*, the type genus of Ctenodrillidae, is a part of Cirratulidae (Bleidorn, Vogt & Bartolomaeus, 2003; Weidhase, Bleidorn & Simon, 2016; Magalhães, Linse & Wiklund, 2017), Ctenodrillidae remains unsynonymised. Further, according to the molecular analyses, monophyly of these four genera is questioned (Weidhase, Bleidorn & Simon, 2016; Magalhães, Linse & Wiklund, 2017). The analyses indicated that *Dodecaceria*, *Ctenodrilus*, and *Raricirrus* form a clade, while other cirratulids form another clade. Molecular data of *Aphropharynx* and *Raphidrilus* has never been reported.

Several specimens of cirratuliform polychaetes lacking dorsal tentacles were collected during the survey of polychaetes in Japanese waters. In this study, we describe the specimens as four new species and provide the phylogenetic tree based on two gene sequences. This is the first report of *Raphidrilus* and *Raricirrus* from Japan.

## Materials and Methods

Specimens of *Ctenodrilus* were collected on 17^th^ July 2020 in intertidal to subtidal areas at Tengan-sanbashi (26°28′04″N, 127°49′32″E), Okinawa-jima Island, Japan. Specimens of *Raphidrilus* were collected on 17^th^ July 2020 in intertidal to subtidal areas, Akasaki beach (26°28′04″N, 127°49′32″E) (collected with *Actaedrilus okinawaensis* Jimi, Fujimoto & Imura, 2020), Okinawa-jima Island and on 26^th^ July 2019 under intertidal rocks, Misaki, Japan (35°09′36″N, 139°36′41″E). Specimens of *Raricirrus* were collected on 12^th^ October 2014 from a whale ulna kept in an aquarium. The ulna was collected from a humpback whale carcass off Atami (35°04′29″N, 139°07′34″E, at a depth of 399 m) during a deep-sea research cruise using the ROV *Hyper-Dolphin* (cruise number: NT13-06, dive number: HD#1501) on March 26, 2013. The carcass was stranded and deployed in the bay on December 3, 2011. For morphological observation, the specimens were fixed in 10% formalin–seawater solution and later washed and preserved in 70% ethanol or fixed and preserved in 70% ethanol. For DNA extraction, the specimens were fixed and preserved in 99.5% ethanol. Fresh specimens were photographed using a digital camera (Nikon D5200). Preserved specimens were examined under stereomicroscopes (Nikon SMZ18 and Nikon ECLIPSE 80i). Drawings are made by using Wacom Cintiq and Clip Studio Paint. Traits that are not visible at the magnification used in the drawing are omitted. Specimens of *Raricirrus* for scanning electron microscopy (SEM) were post-fixed in 2% OsO_4_ for 2 h, dehydrated through a series of ethanol and acetone, critical point dried (BAL-TEC CPD-030), osmium coated (Filgen OPC40), and observed using JEOL JSM-7001F. Specimens of *Ctenodrilus* and *Raphidrilus* for SEM observations were washed in deionized water or PBS buffer and dehydrated in a graded ethanol series, dried in a critical-point dryer (HITACHI HCP-1) using liquid CO_2_, and coated with gold in an ion sputter (HITACHI E-1045). Observations were conducted using a scanning electron microscope (HITACHI S-3000N).

Genomic DNA were extracted from small pieces of the paratypes. DNA extraction, sequencing, alignment, calculating pairwise genetic distances, as well as maximum likelihood (ML) and Bayesian inference (BI) phylogenetic tree construction followed the procedures of Jimi et al. (2021).

Additional sequences of other Cirratuliformia were obtained from GenBank (Table 1). Newly obtained sequences have been deposited in the GenBank (Table 1).

**Table 1:** List of species included in the phylogenetic analysis, together with the respective GenBank accession numbers.

Species	COI	16S	References
Acrocirridae gen. 2 sp. KJO-2009	FJ944536.1	FJ944514.1	Osborn et al. (2009)
Acrocirridae sp. ‘horned bomber’	FJ944534.1	FJ944512.1	Osborn et al. (2009)
Acrocirridae sp. ‘tiburon bomber’	FJ944535.1	FJ944513.1	Osborn et al. (2009)
*Brada* sp. KJO-2011	HQ326970.1	HQ326963.1	Osborn, Haddock & Rouse (2011)
*Brada villosa* CMC01	HQ024270.1	HQ326962.1	Carr et al. (2011)
*Cirratulus* cf. *cirratus* MW-2014	KM083601.1	KT033724.1	Weidhase, Bleidorn & Simon (2016)
*Cirratulus cirratus*	HM417794.1	DQ779609.1	Rousset et al. (2007)
*Cirriformia chicoi*	KM192165.1	KM192189.1	Magalhães et al. (2014)
*Cirriformia tentaculata*	KR916808.1	KT033725.1	Lobo et al. (2016)
*Ctenodrilus japonicus* sp. nov.	MZ647970	MZ663734	This study
*Ctenodrilus pacificus*	KT934277.1	KT934267.1	Magalhães et al. (2016)
*Ctenodrilus* cf. *serratus* MW-2015	KP794931.1	KT033726.1	Weidhase, Bleidorn & Simon (2016)
*Ctenodrilus serratus*	KP794932.1	KT033727.1	Weidhase, Bleidorn & Simon (2016)
*Dodecaceria ater*	KP794933.1	KT033728.1	Weidhase, Bleidorn & Simon (2016)
*Dodecaceria concharum*	DQ209262.1	KT033729.1	Osborn & Rouse, 2008
*Dodecaceria sextentaculata*	KP794935.1	KT033730.1	Weidhase, Bleidorn & Simon (2016)
*Flabelligena* sp. KJO-2008	EU694126.1	EU694113.1	Osborn & Rouse (2008)
*Flabelligera infundibularis*	EU694131.1	EU694112.1	Osborn & Rouse (2008)
*Flabegraviera mundata*	HQ326969.1	HQ326958.1	Osborn, Haddock & Rouse (2011)
*Flota* sp. KJO-2008	EU694129.1	EU694110.1	Osborn & Rouse (2008)
*Macrochaeta clavicornis*	EU791463.1	HQ326957.1	Osborn & Rouse (2008)
*Macrochaeta* sp. KJO-2008	EU694125.1	EU694114.1	Osborn & Rouse (2008)
*Raphidrilus misakiensis* sp. nov.	MZ647971	MZ663735	This study
*Raphidrilus okinawaensis* sp. nov.	MZ647972	MZ663736	This study
*Raricirrus anubis* sp. nov.	–	MZ663733	This study
*Raricirrus beryli*	HE863972	MF414726	Petersen et al. (2012)
*Raricirrus jennae* California wood	MF414724	MF414727	Magalhães, Linse & Wiklund (2017)
*Raricirrus jennae* ESR vent	MF414725	MF414728	Magalhães, Linse & Wiklund (2017)
*Stylarioides* sp.	HQ326971.1	HQ326960.1	Osborn, Haddock & Rouse (2011)
*Swima bombiviridis*	FJ944530.1	FJ944509.1	Osborn et al. (2009)
*Swima tawitawiensis*	FJ944533.1	FJ944511.1	Osborn et al. (2009)
*Teuthidodrilus samae*	FJ944537.1	FJ944515.1	Osborn et al. (2009)
*Timarete caribous*	KM192173.1	KM192193.1	Magalhães et al. (2014)
*Timarete ceciliae*	KM192179.1	KM192196.1	Magalhães et al. (2014)
*Timarete* cf. *punctata* MW-2015	KP794936.1	KT033731.1	Weidhase, Bleidorn & Simon (2016)
*Timarete punctata*	KM192184.1	KM192205.1	Magalhães et al. (2014)

Type specimens were deposited in the National Museum of Nature and Science, Tsukuba (NSMT). The electronic version of this article in Portable Document Format (PDF) will represent a published work according to the International Commission on Zoological Nomenclature (ICZN), and hence the new names contained in the electronic version are effectively published under that Code from the electronic edition alone. This published work and the nomenclatural acts it contains have been registered in ZooBank, the online registration system for the ICZN. The ZooBank LSIDs (Life Science Identifiers) can be resolved and the associated information viewed through any standard web browser by appending the LSID to the prefix http://zoobank.org/. The LSID for this publication is: urn:lsid:zoobank.org:pub:88A729DD-232C-4241-AF17-8C603D86C231. The online version of this work is archived and available from the following digital repositories: PeerJ, PubMed Central SCIE and CLOCKSS.

## Results

**Systematics**

**Genus *Ctenodrilus* Claparède, 1863**

(Japanese name: kushiito-gokai-zoku)

***Ctenodrilus japonicus* sp. nov.**

(New Japanese name: nihon-kushiito-gokai)

(Figures 1–3)

**Figure 1:**
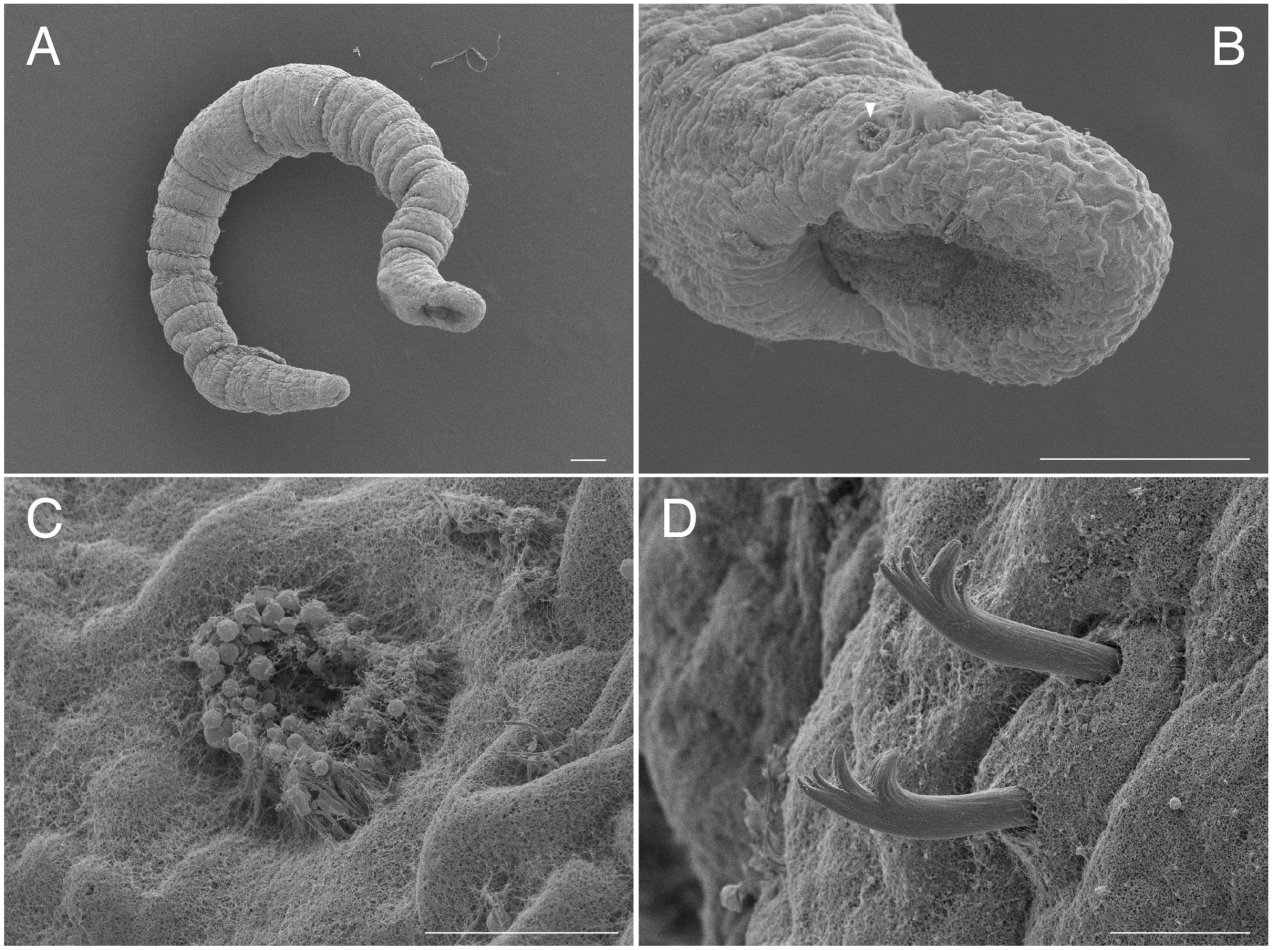
Scanning electron micrographs of *Ctenodrilus japonicus* sp. nov. (NSMT-Pol P-848) (A) whole body; (B) prostomium; (C) nuchal organ; (D) notochaetae, middle segment. Arrow head indicates nuchal organ. Scale bars: A–B, 100 μm; C–D, 10 μm.

**Figure 2:**
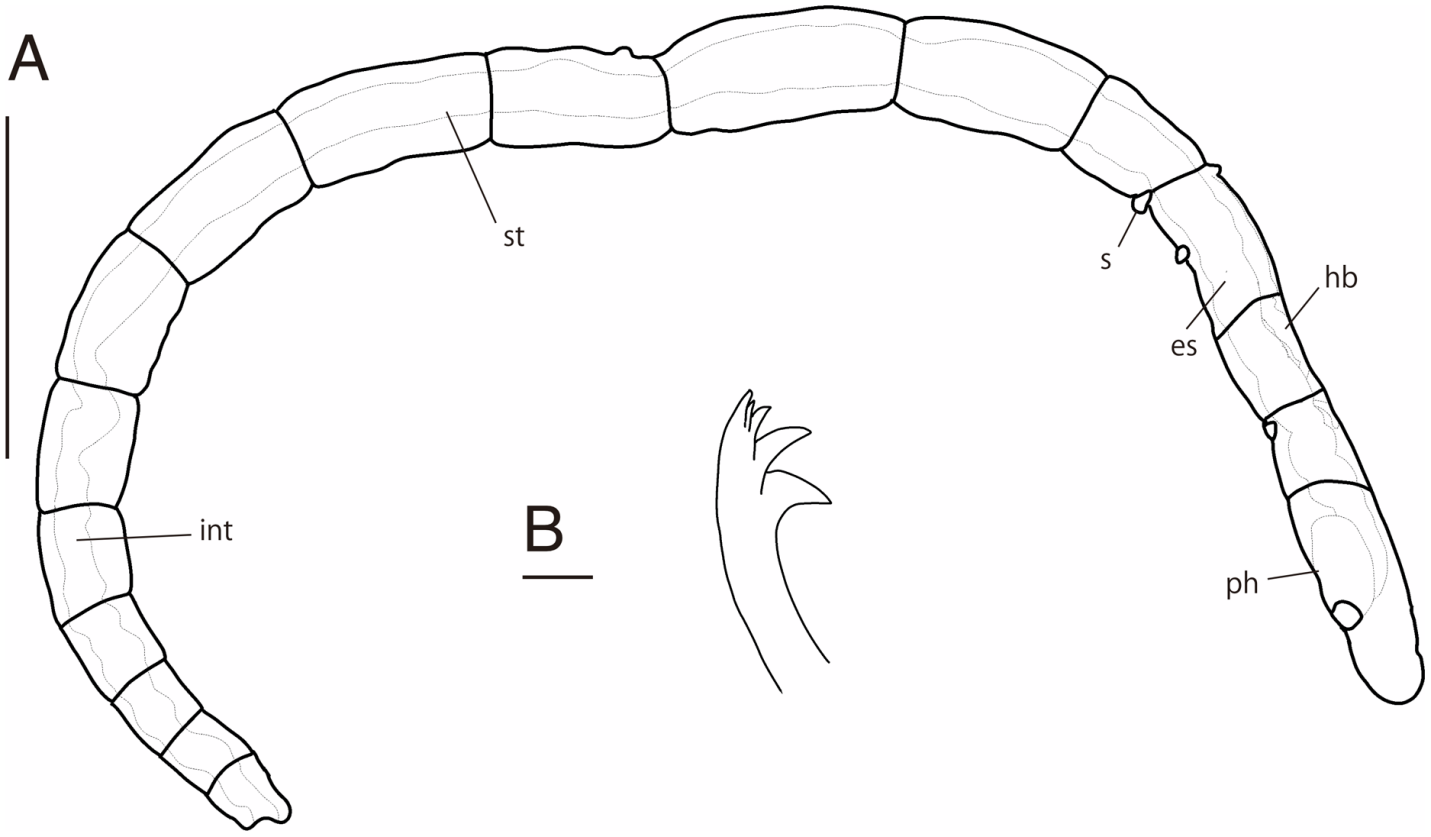
*Ctenodrilus japonicus* sp. nov. (NSMT-Pol H-847) (A) whole view; (B) neurochaeta. Abbreviation: s, scar; es, esophagus; hb, heart body; int, intestine; ph, pharynx; st, stomach. Scale bars: A, 400 μm; B, 10 μm.

**Figure 3:**
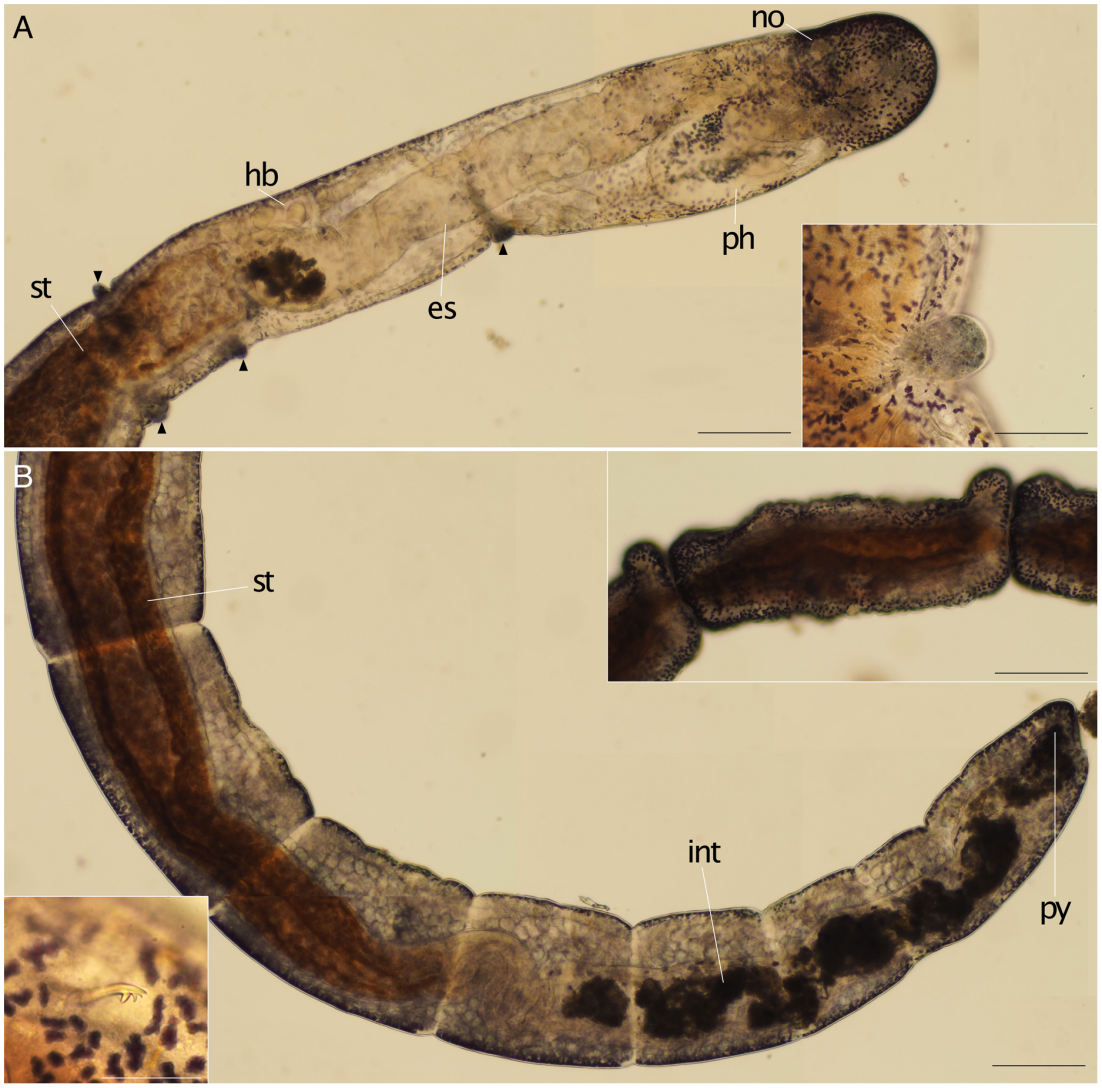
*Ctenodrilus japonicus* sp. nov. (NSMT-Pol H-847) (A) anterior end, inlet showed a scar; (B) posterior end, left inlet showed a neurochaeta, right inlet showed the middle body of paratype. Abbreviation: es, esophagus; hb, heart body; int, intestine; no, nuchal organ; ph, pharynx; py, pygidium; st, stomach. Scale bars: A, B, 200 μm; A (inlet), B (inlet), 100 μm. The figures are made based on several photographs.

**Type material.** Holotype (NSMT-Pol H-847): 1.4 mm long, 0.2 mm wide; sand, Tengansanbashi (26°28′04″N, 127°49′32″E), Okinawa-jima Island, subtidal (0.5 m in depth); collected by NJ and SF. Paratypes five specimens (NSMT-Pol P-848): 1.0–1.3 mm long, 0.2 mm wide; obtained together with the holotype.

**Description of holotype (NSMT-Pol H-847)**. Body cylindrical (Figs. 1A, 2A), 16 chaetigers; first three body segments as long as wide, segments becoming 2–3 times longer than wide from chaetiger 4. Live specimen with black epidermal glands throughout body (Fig. 3), uniformly distributed; yellow pigments also presented on epidermal tissue; whitish in preserved specimens.

Prostomium short, ventrally ciliated, broadly rounded with a pair of postero-lateral rounded nuchal organs (Figs. 1B, 1C); eye-spots absent; nuchal organs in live specimens visible as unpigmented area. Nuchal organs rounded with cilia, about 10 μm diameter. Peristomium not ciliated. Anterior region (prostomium, peristomium, and chaetiger 1) with indistinct borders dorsally and ventrally (Figs. 1A, 1B); clear segment demarcation between chaetigers 1 and 2. Palps and branchiae absent.

Esophagus ciliated to end of chaetiger 2, stomach reddish and ciliated to middle of chaetiger 10. Heart body present from chaetiger 1 to beginning of chaetiger 3 (Fig. 3A). Intestine ciliated to end of the body.

Parapodia biramous. Noto- and neuropodia with 1–4 (mostly 3) multidentate hooks. Number of hooks per segment increasing to chaetiger 10 (2–4 chaetae), from chaetiger 11 decreasing to posterior end (1–3 chaetae). Hooks with 3–4 inner teeth, proximal tooth enlarged and pointed down and other teeth smaller and straighter (Figs. 1D, 2B, 3B). Shape of hooks similar throughout, short, thick and slightly curved (Figs. 1D, 2B, 3B).

Pygidium elongated segment with dorsal anus (Figs. 2A, 3B); anal cilia not seen.

Some scars present between segments (Figs. 2A, 3A), buds absent.

**Variations.** 12–16 chaetigers. In one of the paratypes (NSMT-Pol P-844), middle segments changed shape for asexual reproduction. Anterior margin of the segments is developed.

**Etymology.** The name is derived from the distribution of this new species. The specific name is a noun in the genitive case.

**Distribution.** Interstitial sand of Okinawa-jima, Okinawa, at a depth of 0.5 m.

**Remarks.** *Ctednodrilus japonicus* **sp. nov.** can be distinguished from the other members of the genus by the following features: (*i*) heart body from chaetiger 1 to the beginning of chaetiger 3, (*ii*) body with dark black and yellow spots, (*iii*) esophagus present to end of chaetiger 2, (*iv*) stomach present to the middle of chaetiger 10, and (*v*) 1–4 hooks with 3–4 inner teeth present in noto- and neuropodia. This species most resembles *C*. *pacificus* Magalhães et al., 2016 in having dark black spots and 1–4 hooks with 3–5 inner teeth in noto- and neuropodia. The new species has a heart body extending to the beginning of chaetiger 3 and the stomach extending to the middle of chaetiger 10, while *C*. *pacificus* has a heart body extending to the middle of chaetiger 3 and a stomach extending to the chaetiger 6–8. *Ctenodrilus japonicus* sp. nov. is closest to *C. pacificus* in terms of 565 bp of COI sequences; it was 12.3 % in K2P. From Japanese waters, Sudzuki & Sekiguchi (1972) described *Ctenodrilus serratus limulicolus* from an aquarium in Shimoda Marine Research Center. However, Wilfert (1973) concluded that the subspecies from Japan is not valid because of the variations in traits of the subspecies fall within the range of variation of those traits within *C*. *serratus* (Schmidt, 1857). The new species can be distinguished from *C*. *serratus limulicolus* (Sudzuki & Sekiguchi, 1972), by having a ciliated stomach and 1–4 hooks with 3–5 inner teeth in the noto- and neuropodia. *Ctenodrilus serratus limulicolus* has a non-ciliated stomach and 1–3 hooks with 3–5 inner teeth and 2–3 outer teeth in the noto- and neuropodia.

**Genus *Raphidrilus* Monticelli, 1910**

(New Japanese name: era-kushiito-gokai-zoku)

Type species: *Raphidrilus nemasoma* Monticelli, 1910

**Diagnosis (emended after Magalhães, Bailey-Brock & Davenport, 2011)**

Cirratulidae with peristomium obviously delimited from the prostomium and first achaetous segment both dorsally and ventrally; nuchal organs shallow depressions with cilia; 1–2 dorsally biannulated achaetous segments between peristomium and first chaetiger; posterior end indistinct from posterior segments. Heart body always present from chaetiger 4. Serrate capillaries throughout; more abundant anteriorly. Pectinate neurochaetae absent or present. Reproduction sexual and asexual.

**Remarks**

The genus *Raphidrilus* was characterized by having only capillary chaetae and absence of other type chaetae (Magalhães, Bailey-Brock & Davenport, 2011). However, *Raphi*. *misakiensis* sp. nov., with capillary chaetae and pectinate chaetae similar to those seen in *Raricirrus*, forms a clade with *Raphi*. *okinawaensis* sp. nov. that has typical characters of *Raphidrilus*. They shared some characters (*e.g*., absence of simple curved spine, ability of viviparous reproduction) unlike *Raricirrus*, and thus, we concluded that the new species *Raphi*. *misakiensis* should be treated as a member of *Raphidrilus* and emended the diagnosis of *Raphidrilus*.

***Raphidrilus misakiensis* sp. nov.**

(New Japanese name: Misaki-era-kushiito-gokai)

(Figures 4–6)

**Figure 4:**
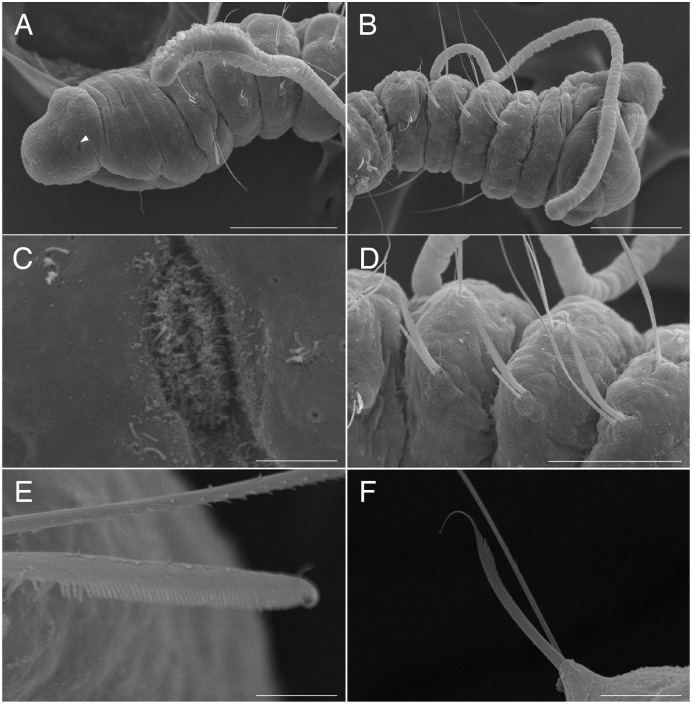
Scanning electron micrographs of *Raphidrilus misakiensis* sp. nov. (NSMT-Pol P-850) (A) anterior end; (B) anterior end of another specimen; (C) nuchal organ; (D) chaetigers 1–3; (E) neurochaetae of anterior segment; (F) neurochaetae of posterior segment. Arrow head indicates nuchal organ. Scale bars: A, B, 100 μm; C, 5 μm; D, 50 μm; E, 5 μm; F, 10 μm.

**Figure 5:**
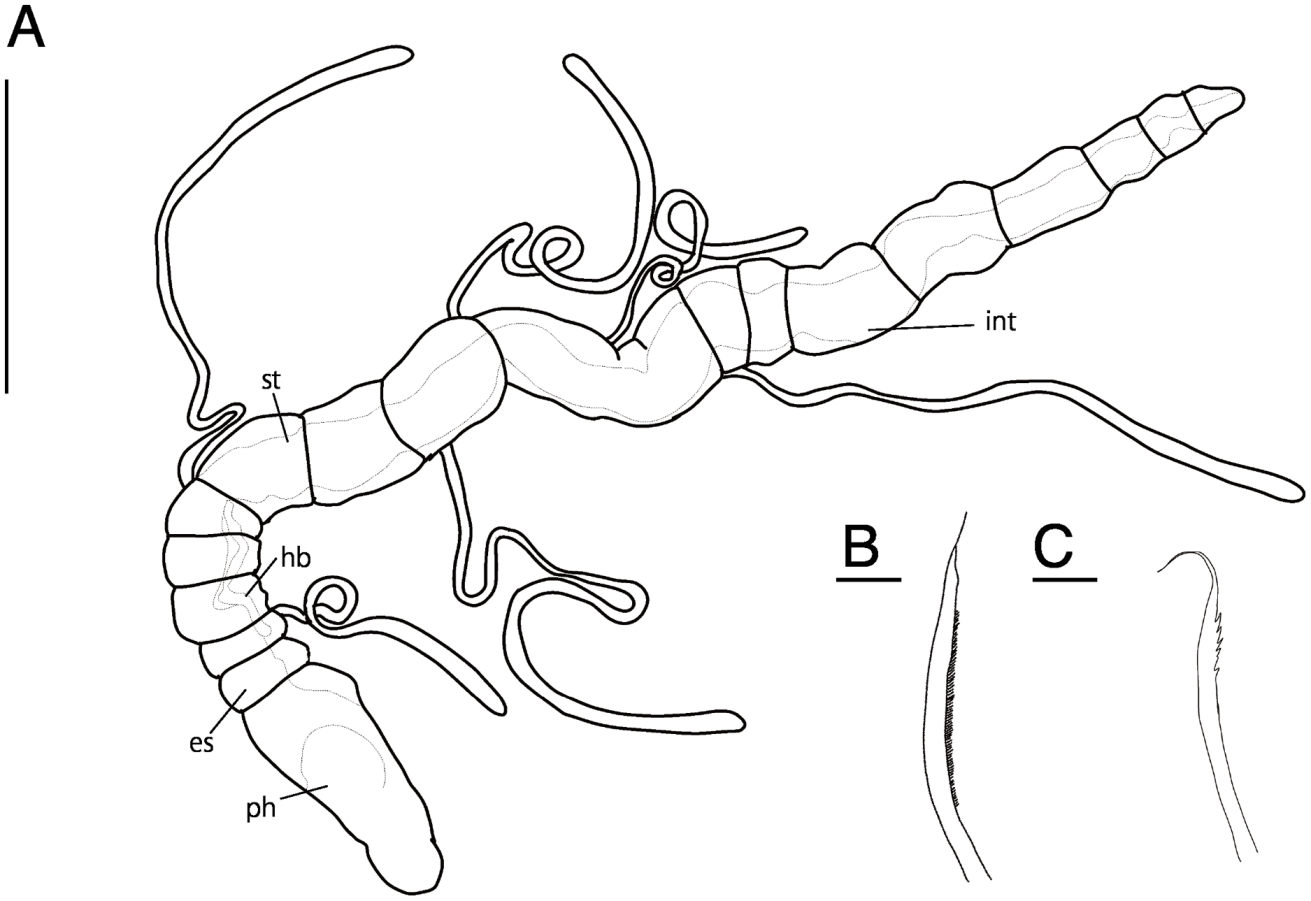
*Raphidrilus misakiensis* sp. nov. (NSMT-Pol H-849) (A) whole view; (B) pectinate notochaeta; (C) pectinate neurochaeta. Abbreviation: es, esophagus; hb, heart body; int, intestine; ph, pharynx; st, stomach. Scale bars: A, 500 μm; B, C, 10 μm.

**Figure 6:**
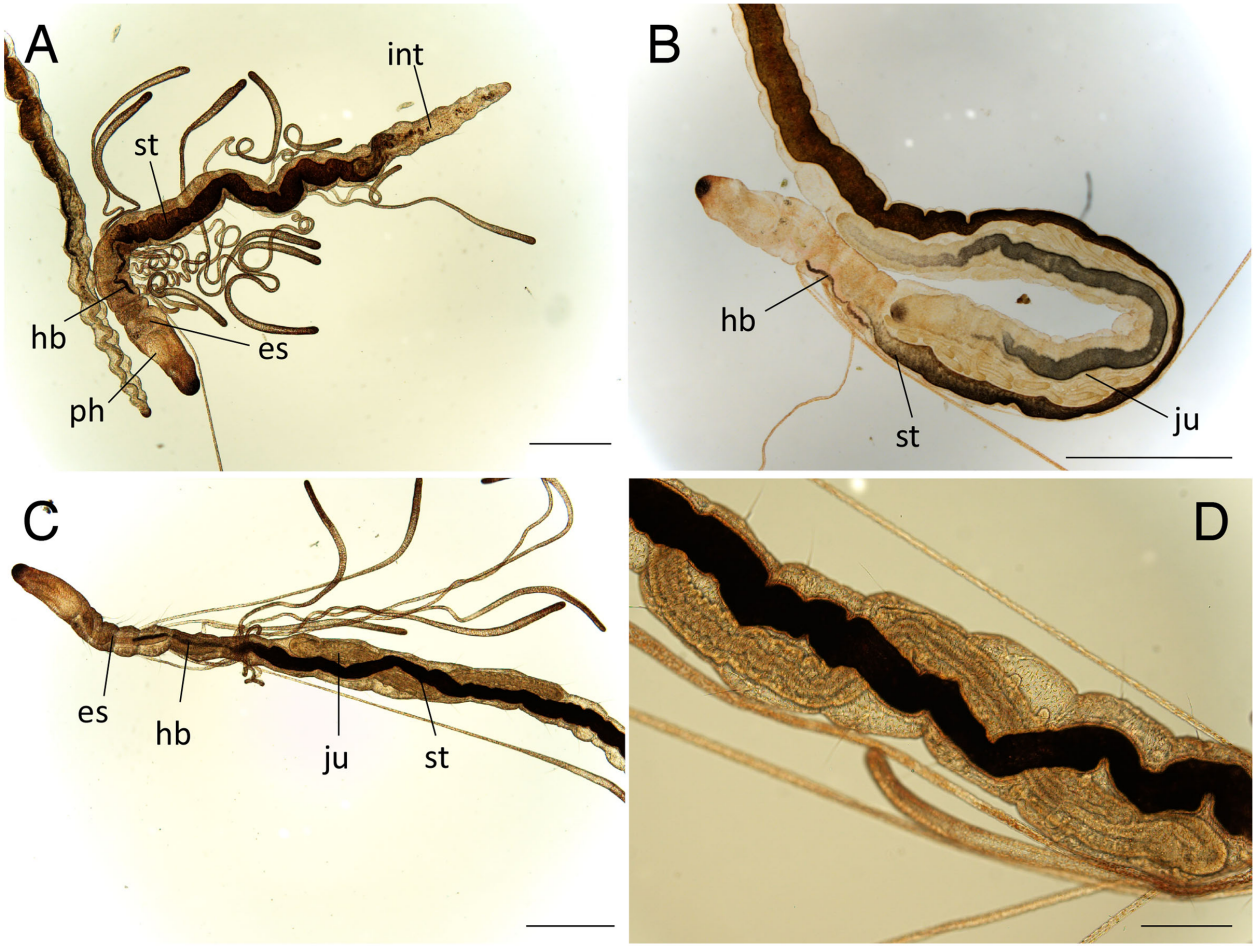
*Raphidrilus misakiensis* sp. nov. (A, NSMT-Pol H-849; B–D, NSMT-Pol P-850) (A) whole body, (B) anterior end, (C) anterior end, (D) enlarged view of juvenile. Scale bars: A–C, 500 μm; D, 200 μm. Abbreviation: es, esophagus; hb, heart body; int, intestine; ju, juvenile; no, nuchal organ; ph, pharynx; st, stomach.

**Type material.** Holotype (NSMT-Pol H-849): 2.0 mm long, 0.2 mm wide; Araihama Beach (35°09′36″N, 139°36′41″E), Misaki, intertidal (under rocks); collected by TM. Paratypes five specimens (NSMT-Pol P-850): 1.5–2.0 mm long, 0.2 mm wide; obtained together with the holotype.

**Description of holotype (NSMT-Pol H-849).** Body cylindrical, 24 chaetigers; first four chaetigers as long as wide (Figs. 4A, 4B), segments becoming 2–3 times longer than wide from chaetiger 5 (Figs. 4A, 4B, 5A). Live specimen orange transparent (Figs. 6A–6C); orange in preserved specimens.

Prostomium short (Figs. 4A, 4B), not ciliated ventrally, broadly rounded with a pair of postero-lateral rounded nuchal organs (Figs. 4A, 4C); eye-spots absent; black pigmentation present anteriorly (Figs. 6A–6C). Nuchal organs rounded with cilia, about 10 μm diameter (Fig. 4C). Peristomium not ciliated. Separation of prostomium and peristomium distinct dorsally and ventrally, indistinct laterally. Separation of peristomium and achaetous segment distinct dorsally, laterally, and ventrally. Separation of achaetous segment and chaetiger 1 distinct dorsally, laterally, and ventrally. Clear segment demarcation between chaetigers 1 and 2. Palps absent. Branchiae ciliated (Figs. 4A, 4B, 4D), brownish in distal area (Fig. 6A), present on chaetiger 5.

Esophagus ciliated to end of chaetiger 5, stomach transparent and ciliated to end of chaetiger 10. Heart body present from middle of chaetiger 3 to end of chaetiger 5 (Figs. 6A–6C). Intestine ciliated to end of body.

Parapodia biramous (Fig. 4D). Notopodia with 1–4 serrated capillary chaetae. First three chaetigers of neuropodia with a pectinate chaeta and a serrated capillary chaeta (Figs. 4B, 4E, 5B). Neuropodia of chaetiger 4 and following chaetigers with a short pectinate chaeta and a serrated capillary chaeta (Figs. 4F, 5C). Last three chaetigers having short pectinate chaetae only in notopodia and neuropodia.

Pygidium elongated segment with dorsal anus; anal cilia not seen.

**Variations.** 20–24 chaetigers. Eye spots present in juvenile. Branchiae present on chaetigers 5–12. One of the paratypes with neuropodial pectinate chaetae in chaetiger 1 and short pectinate chaetae in subsequent chaetigers (Fig. 4A). Last 3–6 chaetigers have short pectinate chaetae only in notopodia and neuropodia. One to two juveniles seen in the body (Figs. 6B, 6D).

**Etymology.** The name is derived from the distribution of this new species. The specific name is a noun in the genitive case.

**Distribution.** Around Misaki (Araihama beach, Moroiso beach, and Douami beach), Japan, intertidal, under the rocks or in the red algae.

**Remarks.** *Raphidrilus misakiensis* **sp. nov.** is the only *Raphidrilus* with pectinate chaetae which distinguishes it from the other congeners. The new species can also be discriminated from the member of *Raricirrus* by the absence of simple curved spines and the occurrence of viviparous reproduction. *Raphidrilus misakiensis* sp. nov. is closest to *R. okinawaensis* sp. nov. in terms of 581 bp of COI sequences; it was 21.3 % in K2P.

***Raphidrilus okinawaensis* sp. nov.**

(New Japanese name: Okinawa-era-kushiito-gokai)

(Figures 7–9)

**Figure 7:**
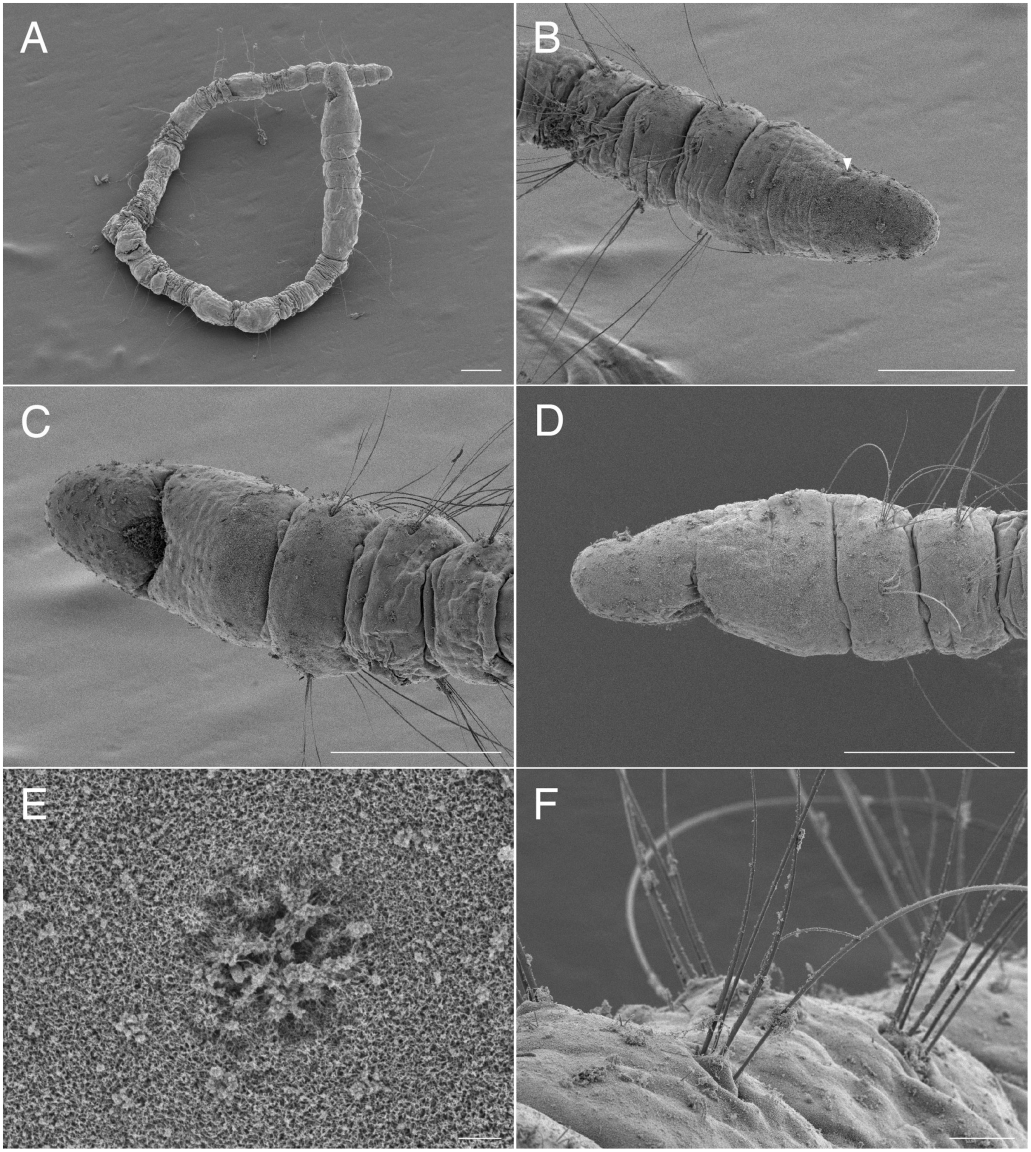
Scanning electron micrographs of *Raphidrilus okinawaensis* sp. nov. (NSMT-Pol P-852) (A) whole body; (B) anterior end of another specimen, dorsal view; (C) anterior end, ventral view; (D) anterior end, lateral view; (E) nuchal organ; (F) noto- and neurochaetae of middle segment. Arrow head indicates nuchal organ. Scale bars: A–D, 100 μm; E, 1 μm; F, 10 μm.

**Figure 8:**
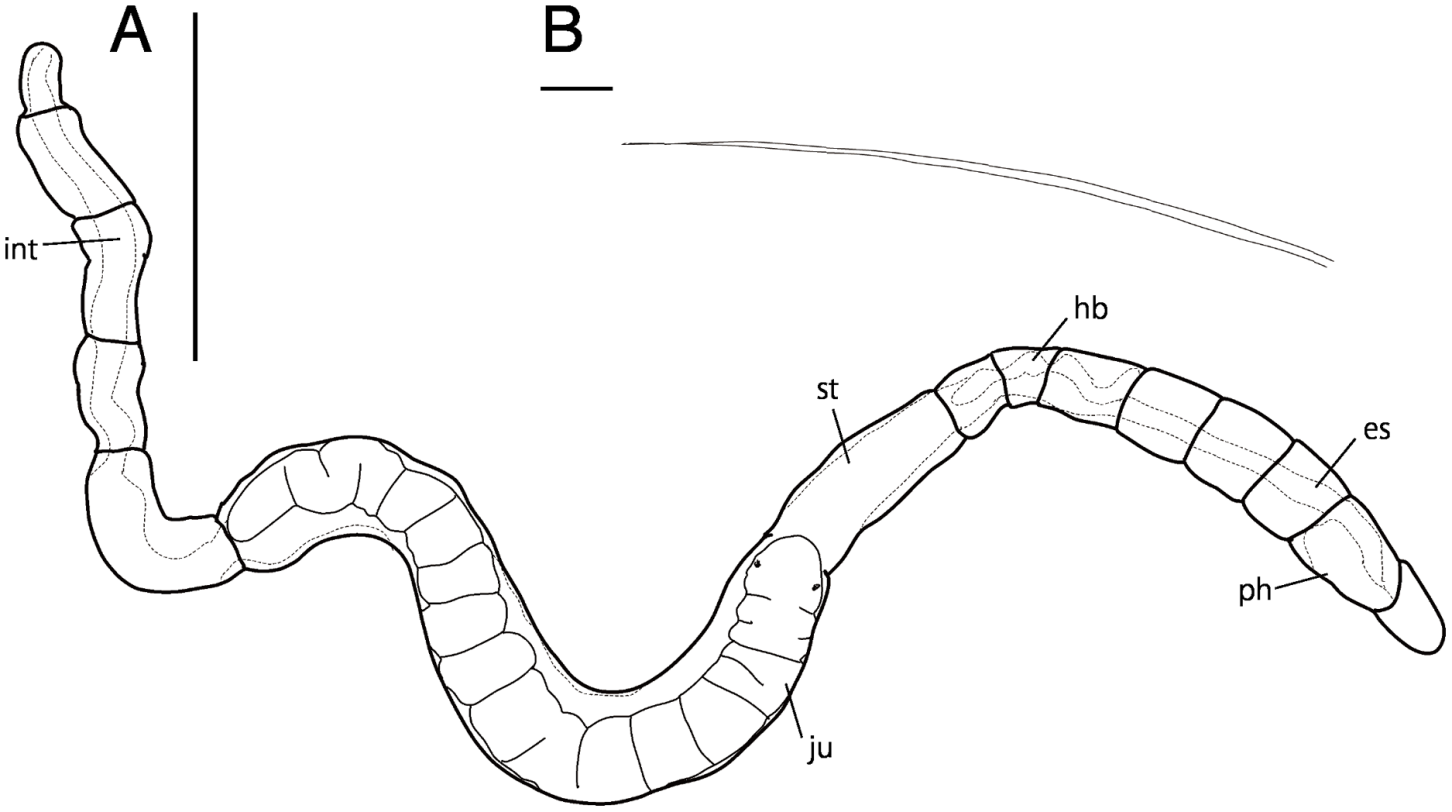
*Raphidrilus okinawaensis* sp. nov. (NSMT-Pol H-851) (A) whole view, (B) neurochaeta. Abbreviation; es, esophagus; hb, heart body; int, intestine; ju, juvenile; ph, pharynx; st, stomach. Scale bars: A, 500 μm; B, 10 μm.

**Figure 9:**
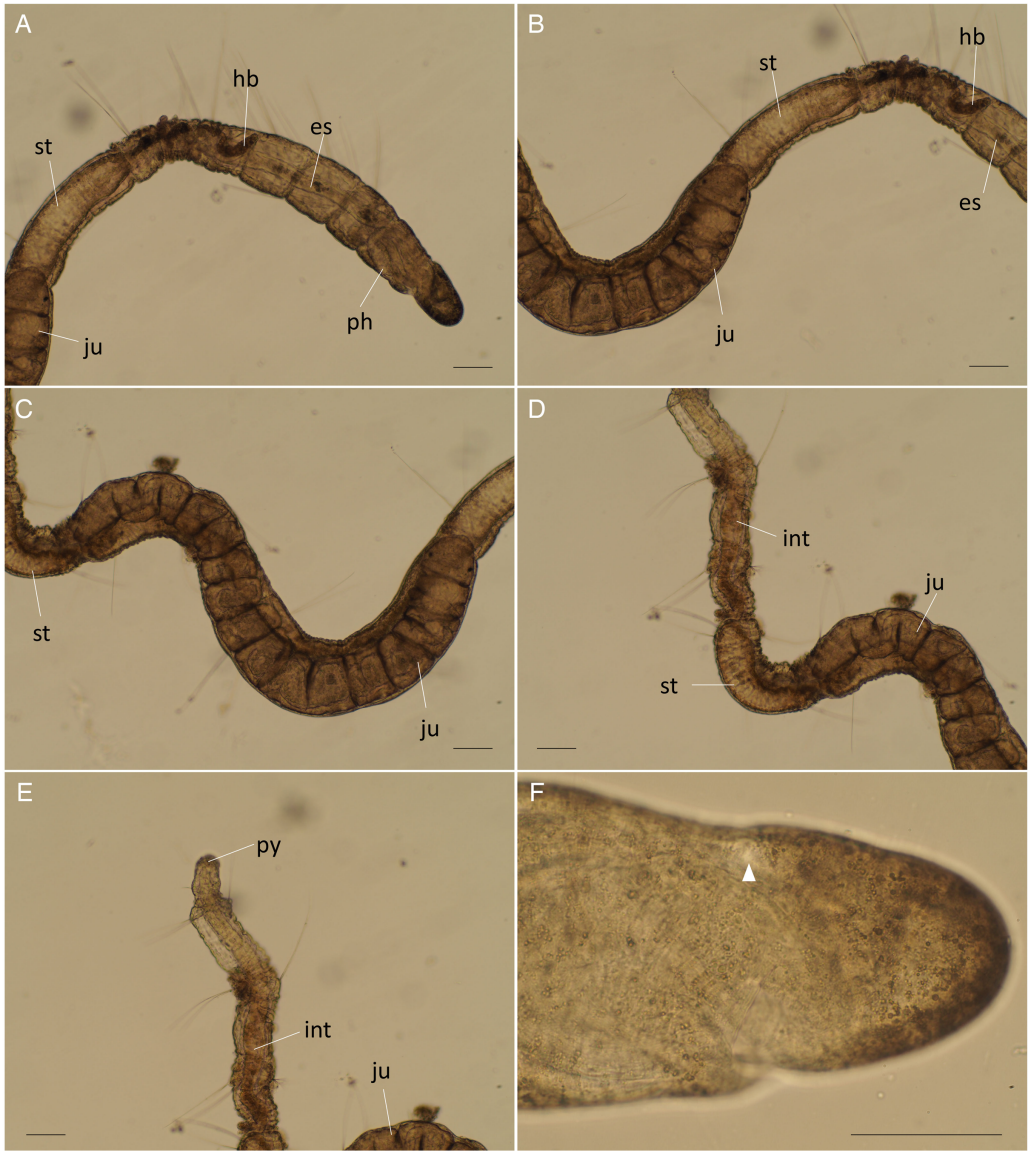
*Raphidrilus okinawaensis* sp. nov. (NSMT-Pol H-851) (A) anterior end; (B–D) middle segments; (E) posterior end; (F) enlarged view of anterior end. Abbreviation: es, esophagus; hb, heart body; int, intestine; ju, juvenile; no, nuchal organ; ph, pharynx; py, pygidium; st, stomach. Scale bars: A–F, 100 μm.

**Type material.** Holotype (NSMT-Pol H-851): 1.7–2 mm long, 0.3 mm wide; Akasaki Beach (26°28′04″N, 127°49′32″E), Okinawa, subtidal (0.5 m in depth); collected by NJ and SF. Paratypes five specimens (NSMT-Pol P-852): 1.7–2 mm long, 0.3–0.4 mm wide; obtained together with the holotype.

**Description of holotype (NSMT-Pol H-851).** Body cylindrical (Figs. 7A, 8A), 21 chaetigers; first four chaetigers as long as wide, segments becoming 2–3 times longer than wide from chaetiger 5. Transparent in living and preserved specimens (Fig. 9).

Prostomium short (Figs. 7B–7D, 8A), not ciliated ventrally (Fig. 7C), broadly rounded with a pair of postero-lateral rounded nuchal organs (Fig. 7E); eye-spots absent; black pigmentation present anteriorly (Figs. 9A, 9F). Nuchal organs rounded with cilia, about 10 μm diameter (Figs. 7E, 9F). Peristomium not ciliated (Figs. 8C, 8D). Separation of prostomium and peristomium indistinct dorsally and ventrally. Separation of peristomium and achaetous segment indistinct dorsally, laterally, and ventrally. Separation of achaetous segment and chaetiger 1 distinct dorsally, laterally, and ventrally. Clear segment demarcation between chaetigers 1 and 2. Palps absent. Branchiae ciliated, present on chaetigers 5.

Esophagus ciliated to end of chaetiger 1 (Figs. 7A, 7B), stomach ciliated to middle of chaetiger 6 (Figs. 7B–7D). Heart body present from beginning of chaetiger 4 to end of chaetiger 5 (Figs. 7A, 7B, 8A). Intestine ciliated to end of body (Figs. 7D, 7E).

Parapodia biramous (Fig. 7F). Notopodia with 5–7 serrated capillary chaetae. Neuropodia with 4–7 serrated capillary chaetae (Figs. 7F, 8B), without pectinate chaetae. Number of notochaetae and neurochaetae decrease posteriorly (2–3 chaetae).

Pygidium elongate segment with dorsal anus; anal cilia not seen.

One juvenile is found within body (Figs. 8A, 9A–9E).

**Variations.** 19–24 chaetigers. One to two juveniles are found in body. Eyespots present in juvenile. Branchiae present on chaetiger 5–12.

**Etymology.** The name is derived from the distribution of this new species. The specific name is a noun in the genitive case.

**Distribution.** Only known from the type locality, the Akasaki beach, Okinawa, Japan, subtidal sands.

**Remarks.** The new species can be distinguished from other congeners by the following characters: (*i*) peristomium and achaetous segment without subannulation; (*ii*) heart body spanning from chaetigers 4 to 5. Other known species of *Raphidrilus* have annulations on peristomium and achaetous segment. Heart body of other species are distributed in chaetiger 4 only (*Raphi*. *nemasoma* Monticelli, 1910), chaetigers 3–4 (*Raphi*. *hawaiiensis* Magalhães, Bailey-Brock & Davenport, 2011), chaetigers 4–7 (*Raphi*. *harperi* Magalhães, Bailey-Brock & Davenport, 2011).

***Raricirrus* Hartman, 1961**

(New Japanese name: ito-nashi-kushiito-gokai-zoku)

***Raricirrus anubis* sp. nov.**

(New Japanese name: hakamori-kushiito-gokai)

(Figures 10–12)

**Figure 10:**
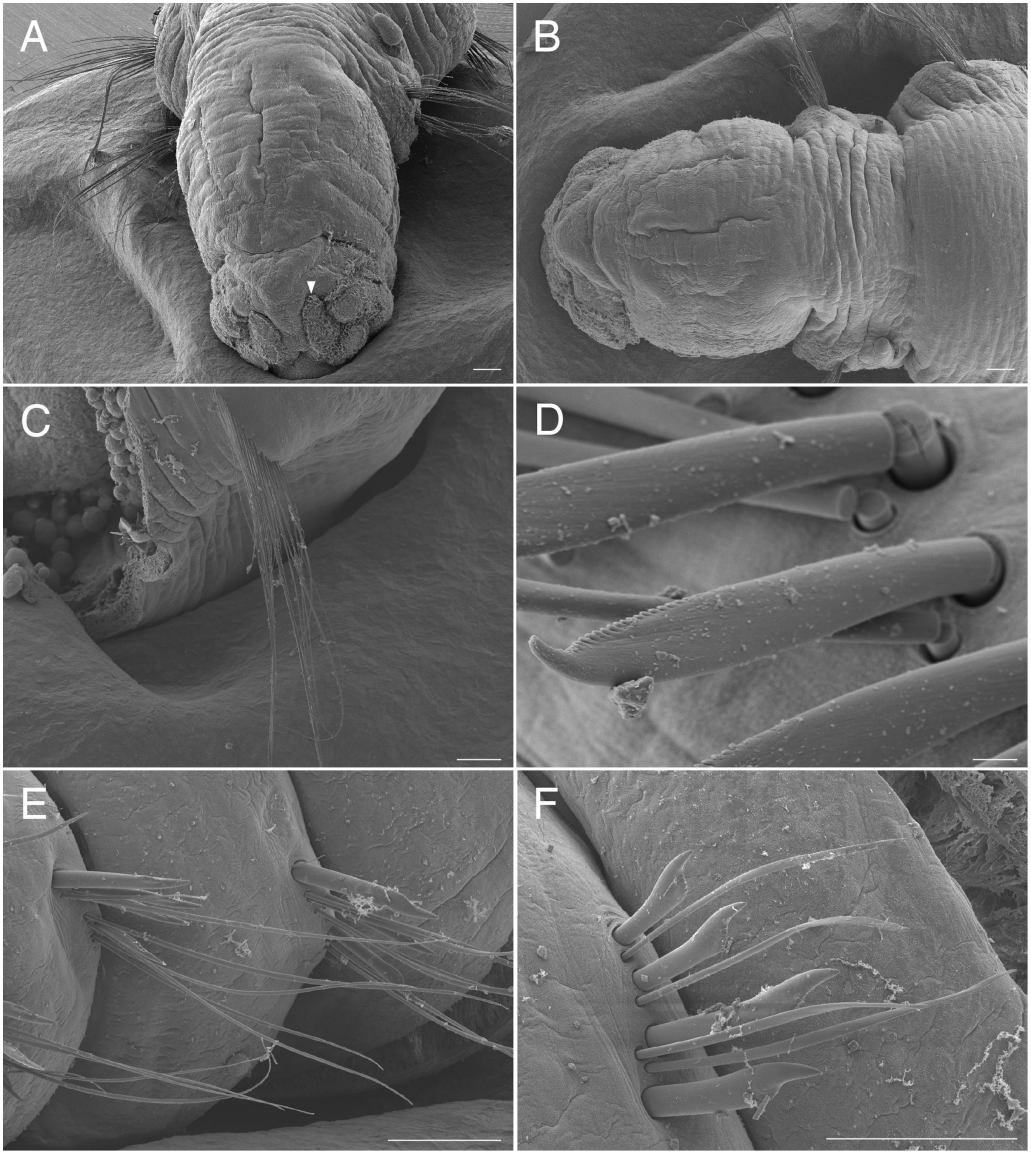
Scanning electron micrographs of *Raricirrus anubis* sp. nov. (NSMT-Pol P-854) (A) anterior end; (B) anterior end, dorsal view; (C) notochaetae of middle segment; (D) neurochaetae of middle segment; (E) notochaetae of posterior segments; (F) neurochaetae of posterior segment. Arrowhead indicates nuchal organ. Scale bars: A–C, 100 μm; D, 5 μm; E–F, 100 μm.

**Figure 11:**
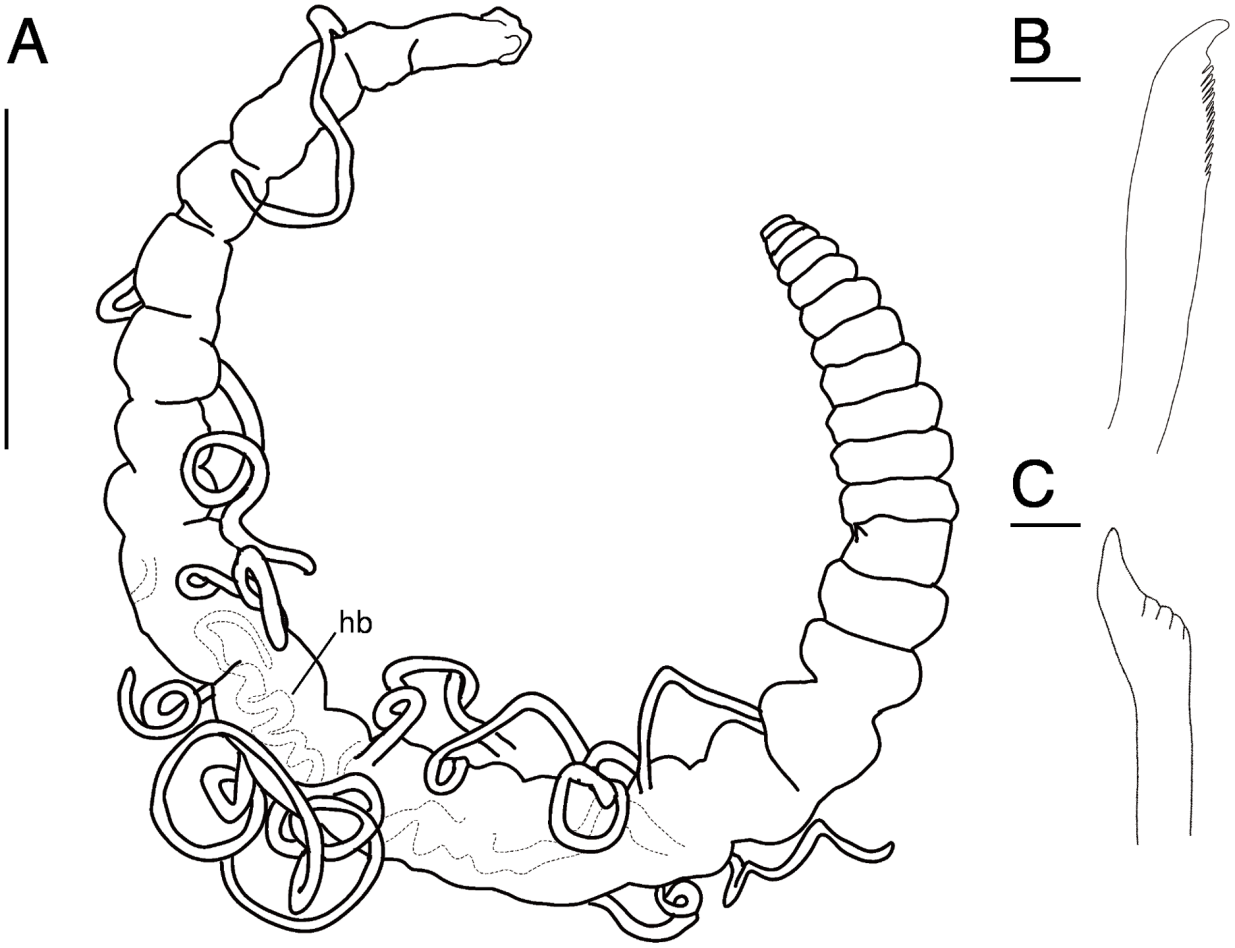
*Raricirrus anubis* sp. nov. (NSMT-Pol H-853) (A) whole body, lateral view; (B) neurochaetae of middle segment; (C) neurochaetae of posterior segment. Abbreviation: hb, heart body. Scale bars: A, 5 mm; B, C, 20 μm.

**Figure 12:**
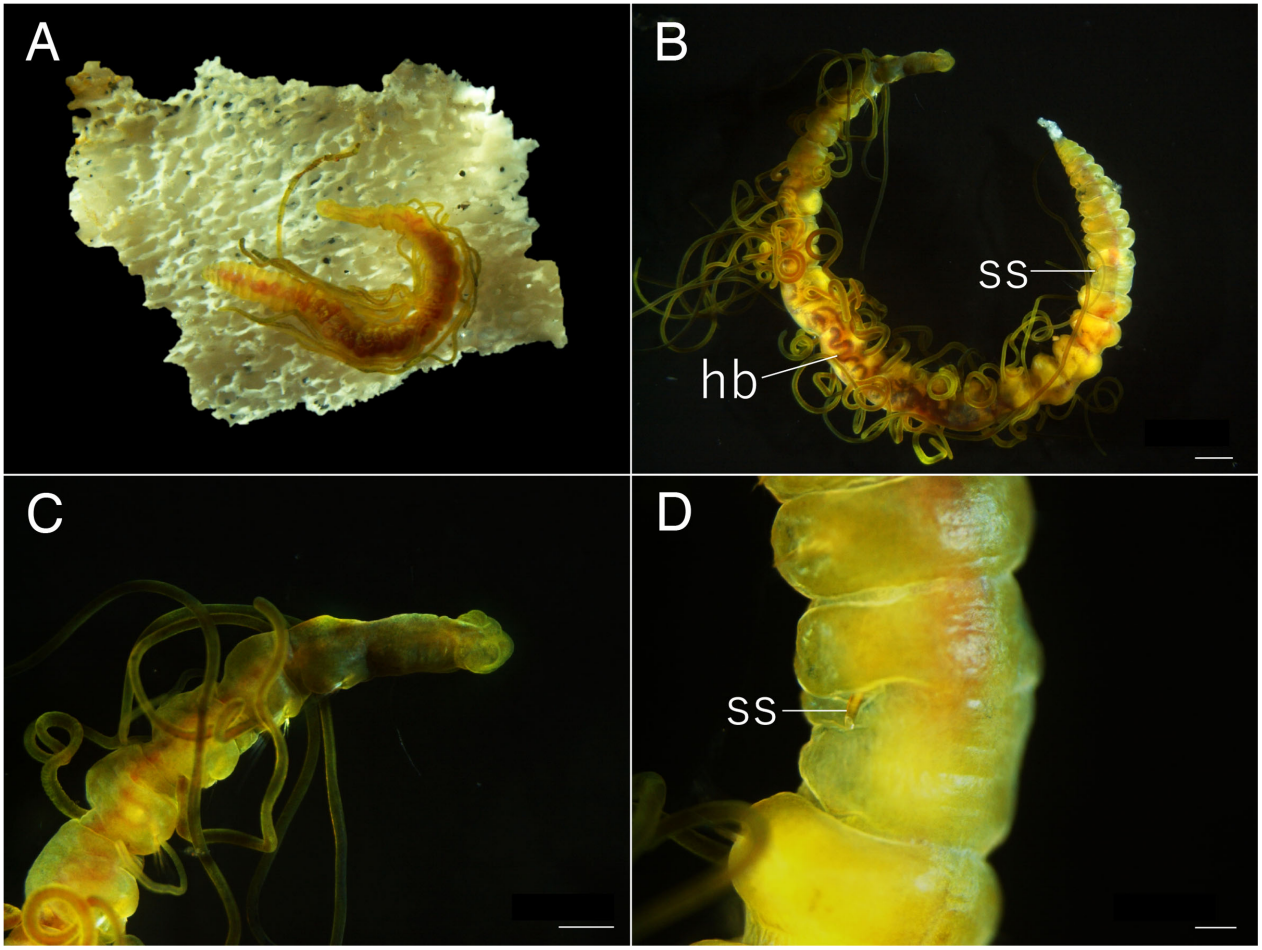
*Raricirrus anubis* sp. nov. (A, NSMT-Pol P-854; B–D, NSMT-Pol H-853) (A) whole body, dorsal view; (B) whole body. Dorsolateral view; (C) anterior end; (D) enlarged view of a simple spine. Abbreviation: hb, heart body; ss, simple spine. Scale bars: B, 1 mm; C, 500 μm; D, 200 μm.

**Type material.** Holotype (NSMT-Pol H-853): 17 mm long, 1 mm wide; aquarium of JAMSTEC, whale bones (recovered from Sagami Bay, Japan; 35°04′29″N, 139°07′34″E); collected by NJ. Paratypes five specimens (NSMT-Pol P-854): 15–29 mm long, 1 mm wide; obtained together with the holotype.

**Description of holotype (NSMT-Pol H-853).** Body cylindrical (Figs. 10A, 11A, 12A, 12B), 29 chaetigers. Yellow in life (Fig. 12). Preserved specimens white or black. Prostomium short (Figs. 10A, 10B, 12C), with nuchal organ ventrally and anterior end ciliated (Fig. 10A). Eye-spots absent. Separation of prostomium and peristomium indistinct dorsally and distinct ventrally; separation of peristomium and achaetous segment indistinct dorsally and distinct ventrally; separation of achaetous segment and chaetiger 1 indistinct dorsally and distinct ventrally; clear segment demarcation between chaetigers 1 and 2. Palps absent. Branchiae ciliated, present on chaetiger 5–15.

Esophagus ciliated to end of chaetiger 1. Heart body present in chaetigers 9–14 (Fig. 12B), reddish brown colour tube (Fig. 12B).

Notopodia have three types of chaetae: short capillary chaeta (Fig. 10A); long capillary chaeta (Fig. 10C); and coarsely serrated chaeta. Neuropodia have three types of chaetae: short pectinate chaetae (Figs. 10D, 11B); long pectinate chaetae; coarsely serrated chaetae (Figs. 10E, 10F, 11C). Capillary chaetae of neuropodia present from chaetiger 1 to following chaetigers. Teeth of neuropodial long and short pectinate chaetae dense and directed obliquely upward. Teeth of notopodial and neuropodial coarsely serrated chaetae poorly developed and directed upward. Paired yellow spines occur in chaetiger 8–10 from last chaetiger (Figs. 12B, 12D), larger than other chaetae. Pygidium rounded, no cirri. All specimens have gametes in some middle chaetigers (around chaetigers 5–20) (Fig. 12B).

**Variations.** 29–30 chaetigers. Branchiae present on chaetigers 1–18. Some specimens regenerated.

**Etymology.** “Anubis” is a Greek name of an ancient Egyptian god that was a grave keeper. This worm lived around whale skeletons perhaps as a grave keeper. The specific name is a noun in the nominative case.

**Distribution.** Only known from whale skeletons of the type locality, at a depth of 399 m.

**Remarks.** The new species can be discriminated from its congeners by the following characters: (*i*) heart body present in chaetigers 9 to 14; (*ii*) capillary chaetae of neuropodia present from chaetiger 1; (*iii*) simple curved spines present. The heart body of other species are distributed in chaetigers 9–12 (*Rari*. *maculatus* Hartman, 1961), chaetigers 9–21 (*Rari*. *beryli* Petersen & George, 1991), chaetigers 4–11 (*Rari*. *variabilis* Dean, 1995), chaetigers 10–16 (*Rari*. *jennae* Magalhães, Linse & Wiklund, 2017), chaetigers 2–15 (*Rari*. *arcticus* Buzhinskaja & Smirnov, 2017). Capillaries of neuropodia 1 are absent in most of the species except *Rari*. *jennae*. Simple curved spines absent in *Rari*. *beryli* and *Rari*. *jennae*. *Raricirrus anubis* sp. nov. is closest to *R. pacificus* in terms of 261 bp of 16S rRNA sequences; it was 6.4 % in K2P (we could not determine the COI sequence of *R*. *anubis* sp. nov.).

**Phylogenetic analysis**

**Figure 13:**
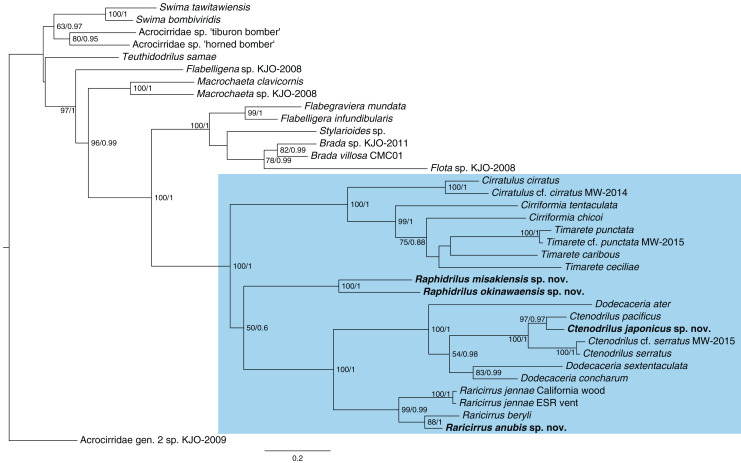
Maximum-likelihood phylogenetic tree of Cirratuliformia based on COI and 16S sequences. Bootstrap support values higher than 50% are indicated on each branch (left). Posterior probability values are also indicated (right).

The topologies (Fig. 13) recovered by ML and BI analyses were identical. *Ctenodrilus japonicus* sp. nov. and *C*. *pacificus* Magalhães et al., 2016 formed a single clade with 97% of bootstrap support in ML and 0.97 posterior probability in BI. *Raphidrilus misakiensis* sp. nov. and *Raphi*. *okinawaensis* sp. nov. formed a single clade with 100 % of bootstrap support in ML and 1.00 posterior probability in BI. *Raphidrilus* is sister to the *Dodecaceria*–*Ctenodrilus*–*Raricirrus* clade, although they comprise a poorly supported clade (50 % bootstrap support, 0.6 posterior probability). *Raricirrus anubis* sp. nov. and *Rari*. *beryli* formed a single clade with 88 % of bootstrap support in ML and 1.00 posterior probability in BI.

## Discussion

Our phylogenetic analysis confirmed that *Ctenodrilus*, *Raphidrilus*, and *Raricirrus* form a monophyletic clade with cirratulid genera. Species of *Ctenodrilus* form a monophyletic clade and are nested within *Dodecaceria*. These results agree with other previous taxonomic studies with molecular analyses (Magalhães et al., 2016; Magalhães, Linse & Wiklund, 2017; Weidhase, Bleidorn & Simon, 2016). This topology warrants the revision of Ctenodrillidae and Cirratulidae. However, the scope of this study is to describe the four new species from Japan and infer their phylogenetic positions and no broad morphological re-assessment spanning the two families were performed. For this reason, we treated these genera as belonging to Cirratuliformia.

Our study also provided the first molecular data of *Raphidrilus*. The genus is sister to the *Dodecaceria*–*Ctenodrilus*–*Raricirrus* clade. However, the *Raphidrilus*–*Dodecaceria*–*Ctenodrilus*–*Raricirrus* clade is poorly supported by bootstrap supports (50 BS) and posterior probability (0.6 PP). It is unclear what the relationship is between the three clades (1. *Raphidrilus*, 2. *Cirratulus*–*Cirriformia*–*Timarete*, 3. *Dodecaceria*–*Ctenodrilus*–*Raricirrus*) at this level of support. A previous morphology-based study inferred that *Raphidrilus* is close to *Dodecaceria* (Magalhães, 2015). In order to understand the evolutionary history of Cirratuliformia especially adaptation to the interstitial lifestyle, further OTUs and gene regions are needed for constructing robust phylogenetic trees. Especially, broader OTUs covering the cirratulid genera will help solving this problem.

In Japan, *Ctenodrilus serratus limulicolus* (Sudzuki & Sekiguchi, 1972) has been known as the sole *Ctenodrilus* species. Our new species *C*. *japonicus* sp. nov. is clearly separated from the species by several characters. There is no doubt that two species of *Ctenodrilus* inhabit Japan. However, the taxonomic status of *C*. *serratus limulicolus* is still unclear. Topotype sampling and DNA sequences of the species are needed for confirmation of *C*. *serratus limulicolus* validity in the future studies. Magalhães et al. (2016) described *C*. *pacificus* from Hawaii, Pacific Ocean. *Ctenodrilus japonicus* sp. nov. is morphologically most similar to the Hawaiian species, as described in the Remarks. In the phylogenetic tree, *C*. *japonicus* and *C*. *pacificus* form a clade with high support (97 BS/0.97 BP). Besides asexual reproduction by stolons (Scharff, 1887; Schmidt, 1857; Kennel, 1882; Magalhães et al., 2016; this study), *Ctenodrilus* is known to reproduce sexually by viviparity (Halt et al., 2006). Both ways have low dispersal ability and do not allow gene exchange to occur over long distances, which may promote geographical isolation in both species. Although it is not known how the common ancestor arrived in Hawaii, the phylogenetic relationships of *Ctenodrilus* species in different regions may shed light on the process of dispersal and speciation.

This study represents the first record of *Raricirrus* and *Raphidrilus* from Japan. *Raricirrus anubis* sp. nov., like other species of the genus, has been observed in whalebones and is consistent with known habitats (Petersen et al., 2012; Magalhães, Linse & Wiklund, 2017). *Raphidrilus okinawaensis* sp. nov. was collected from interstitial environments of sandy beaches like other species of the genus (Magalhães, Bailey-Brock & Davenport, 2011), but *Raphidrilus misakiensis* sp. nov. was collected from unusual environments such as attached to rocks. *Raphidrilus misakiensis* sp. nov. may have a slightly different ecology from other species of the genus because of its unusual habitat and unusual chaetae. Further field observations are needed.

Discovering an unusual morphological character (presence of pectinate chaetae) in *Raphidrilus misakiensis* sp. nov. led us to modify the genus diagnosis. In Qian & Chia (1989), an undescribed species of *Raphidrilus* was found to have genital spines which Magalhães, Bailey-Brock & Davenport (2011) did not include in the definition of the genus regarding it as a species-specific trait. It is possible that the special trait of *R*. *misakiensis* is also species-specific, but we have included it for now. As the diversity of the genus *Raphidrilus* becomes better known, the definition of the genus will become clearer.

## Conclusions

The genera *Ctenodrilus*, *Raphidrilus*, and *Raricirrus* are a poorly known group in Cirratuliformia. In Japan, there has been only one study on this group, in which almost nothing is known about. Molecular phylogenetic analyses have been carried out in a number of previous studies in which some genera were not included. In this study we describe four new species from Japan and construct a phylogenetic tree using two genes to clarify the phylogenetic relationships within this group. As a result the phylogenetic position of *Raphidrilus* was not clearly determined, therefore further analyses should be required including additional OTUs in the future. This is the first record of *Raphidrilus* and *Raricirrus* from Japan.

## Supplemental Information

10.7717/peerj.13044/supp-1 Supplemental Information 1 DNA sequences.

## Additional Information and Declarations

### Field Study Permissions

The following information was supplied relating to field study approvals (*i.e*., approving body and any reference numbers): Permissions are not required in the sampling localities in the present study.

### Data Availability

The following information was supplied regarding data availability: The raw sequence data are available in the Supplemental File and GenBank: MZ647970 to MZ647972; MZ663733 to MZ663736. All specimens are stored at the National Museum of Nature and Science, Japan (NSMT). Specimens No.: NSMT-Pol H-843, NSMT-Pol P-844, NSMT-Pol H-845, NSMT-Pol P-846, NSMT-Pol H-847, NSMT-Pol P-848, NSMT-Pol H-849, NSMT-Pol P-850.

### New Species Registration

The following information was supplied regarding the registration of a newly described species: Publication LSID: urn:lsid:zoobank.org:pub:88A729DD-232C-4241-AF17-8C603D86C231
*Ctenodilus japonicus:*
urn:lsid:zoobank.org:act:9F7EF214-4F0A-40C9-85AD-26B1D3043868
*Raphidrilus misakiensis:*
urn:lsid:zoobank.org:act:D270E580-F8D9-4FC3-B83F-90B25EF28098
*Raphidrilus okinawaensis:*
urn:lsid:zoobank.org:act:308E1797-9184-40ED-98A5-B7107D7352D9
*Raricirrus anubis:*
urn:lsid:zoobank.org:act:F4C1E4E5-23EC-4C32-AC7C-A4C36A59B4F0.
