# Supplementary material for: Four new species of Ctenodrilus, Raphidrilus, and Raricirrus (Cirratuliformia, Annelida) in Japanese waters, with notes on their phylogenetic position
Source: PeerJ. 2022 Mar 8;10:e13044. doi: 10.7717/peerj.13044 (PMC8916031; doi:10.7717/peerj.13044)
Supplement: Supplemental Information 1 [file peerj-10-13044-s001.docx]

>Ctenodrilus_japonicus_COI

CAAATCATAAAGATATTGGAACCCTATACCTTCTTCTAGGAATCTGATCAGGCCTTATTGGCACGTCATGCAGGCTACTC

ATTCGAATTGAGTTAAGACAGCCTGGCTCATTCCTAGGAAATGACCAAATCTACAACGTCCTAGTCACCGCACACGCTTT

CCTAATAATTTTCTTTATAGTTATACCAGTGTTCGTAGGAGGTTTCGGGAACTGACTAATCCCCCTCATACTGGCAACCC

CAGACATAGCATTCCCACGCCTAAACAACCTAAGACTTTGACTCTTGCCCCCCGCACTCACACTTCTCATCCTCTCAGCC

CTAGTTGAACAGGGAGCAGGGACAGGATGAACCGTCTACCCCCCTCTAGCCGGCTCAATTGCTCACAGAGGCCCATCCGT

AGATCTCGCAATCTTCTCCCTCCACCTAGCCGGTGCCTCATCAATTCTAGGGTCCCTAAACTTCCTTACTACTGCCTACA

ACATGCGACCAGAAACACTAATAGCCGAGCGAATCCCATTATTTGTTTGATCCCTGATCGTAACCGCCGTCCTACTTGTA

CTGTCTCTTCCAGTTCTAGCAGGAGCCATTACAATACTACTAACAGACCGAAACTTAAATTCATCATTCTTCATCCCGGA

AGGAGGAGGAGATCCCGTTCTGTACCAACACCTATTCTGATTTTTTGGTCAC

>Raphidrilus_misakiensis_COI

TATTTGAGCTGGCCTTCTCGGCACATCAATAAGCCTACTTATTCGAATCGAACTTGCCCAGCCTGGCACATTCCTAGGAA

ACGACCAAATTTACAATGTCCTGGTCACAGCCCACGCATTCCTGATAATTTTTTTCTTAGTTATACCAGTCCTAGTAGGT

GGATTTGGGAACTGACTAATCCCCCTAATACTATCTTGCCCAGACATAGCCTTCCCACGAATAAACAACCTCAGATTCTG

ACTTCTTCCTCCCGCTCTACTCCTACTTGTTGCCTCCGCAACAGTTGAACGAGGCGCTGGAACGGGATGAACCGTATACC

CGCCCCTAGCCAGAGTAACTGCCCACAGAGGCCCATCAGTAGATCTTGCAATCTTCTCTCTACACCTTGCAGGAATTTCA

TCAATTCTAGGATCAATTAACTTCATCACCACCTCACGAAACATACGACCAAAAGGGTGTACCGCAGAACGAATTCCCTT

ATTCGTTTGATCACTAATTGTAACCACAATCCTCCTACTCCTATCACTCCCAGTTCTAGCAGGAGCAATCACTATGCTTC

TAACTGATCGAAACTTAAATACATCATTCTTCGACCCAGCAGGGGGCGGAGACCCAATCCTTTACCAACACCTATTCTGA

>Raphidrilus_okinawaensis_COI

ACCTGGATCATTTCTAGGAAATGATCAAATTTATAACGTTTTAGTTACTGCCCACGCATTCTTAATAATCTTCTTTCTAG

TTATACCAATCTTAGTTGGGGGTTTCGGAAACTGACTTATTCCACTTATACTATCCTGCCCGGATATAGCTTTCCCACGT

ATAAACAACTTAAGATTTTGACTTTTGCCCCCCTCTCTCCTACTCTTAATTATATCCGCAATCGTAGAACGAGGTGCAGG

GACCGGTTGAACAGTCTATCCCCCTCTAGCTAGAGTCCCTGCCCATAGAGGCCCCTCAGTAGACCTAGCTATTTTCTCCC

TCCACCTAGCAGGTGTTTCTTCAATTCTAGGCTCAATTAACTTCATCACCACAGCACAAAATATACGACCAAAAGGCTAC

TCCGCCGAACGAATCCCACTCTTCGTTTGATCGCTAATCGTTACAACAATCCTCCTTCTTCTCTCTCTCCCAGTTTTGGC

TGGAGCTATCACTATACTATTGACAGACCGAAATTTAAACACATCCTTTTTTGACCCTGCTGGTGGTGGAGATCCAATTC

TCTACCAACACTTATTCTGAT

>Ctenodrilus_japonicus_16S

AAAAACATTGCCTGTAGATAAACAATTACAGGTACATCCTGCCCAGTGATCCCTCATTCAACGGCCGCGGTACACTGACC

GTGCTAAGGTAGCGCAATTACTTGCCTTTTAATTGAAGGCCCGTACGAACGGACCCACGAAAGCCTAACTGTCTCACAGC

TATAAAAGAAAACTAATCTTTAAGTGAAAAAGCTTAACTAAAATAAAAAGACAAGAAGACCCTGTAGAGCTTAACTCCCA

CAACTATTTCATCCACCTACTTCTGTACACCCTCCCCCTAGTCCAAGAGTTTTGCTGGGGCAGCAAGAGACCAACCAAAA

CGTCTCCTCACAAACCAGACCCTAAGTCCTCCACAAGACCCCTTTATTCCCCCAACGAACCTAAGGAACTTAAAACTTAG

CTACCTCAGGGATAACAGACTAATTTGTCTTAAAAGTCCACATTAACAGACAAGTTTGGCACCTCGATGTTGGCTCAGGA

TCCCCCTAGTGTGCAGCAGCACTATTGGTAAGTATGTTCTCCTTTTAAAATCCTACGTGATC

>Raphidrilus_misakiensis_16S

CAAAAACATTGCCTCTTGATAACCTATAAAAGGTACATCCTGCCCAGTGAACTTTCAACGGCCGCGGTACCCTGACCGTG

CTAAGGTAGCATAATCAACTGCCCTTTAATTAGGGGCCTGTATGAATGGACACACGAAAGCGTAGCTGTCTCAGTGGGAA

ACCTAAAAATTAGCCTCTAAGTGAAGAGACTTAGATTTCACGGCAAGACAAAAAGACCCTGTAGAGCTTCATTTTAAAGC

TAACTTACCACGGCACTTCTGTGTCCCTAGTAAAATTAACCTAAAATTTGGTTGGGGCGACAAAGGACCAAATAAAACGT

CCTTAAATAATAAGACTATAAGTCACCTAAAGACTCATTAGACAACTTGTTTAAGAAGAACCAGCTACCTCAGGGATAAC

AGGCTAATATCCCCTTAAGAGCCCAAATCGACAGGGGAGCTTGGCACCTCGATGTTGGCTTAGGGAACCCGAATTAATGC

AGCAGTTAATAACGGGCGGTTTGTTCAACCGATAATCCCCTACGTGATCTGA

>Raphidrilus_okinawaensis_16S

CAAAAACATTGCCTTCTGCCTAATACATAAAAGGTACATCCTGCCCAGTGAATTTTAAACGGCCGCGGTACCTTGACCGT

GCTAAGGTAGCATAATAGTTTGCCCTTTAATTGGGGGCCTGTATGAACGGACACACGAAAGCGCAACTGTCTCAGCAGAA

CTTATAAAAATTAGCCTCTAAGTGAAGAGACTTAGATTACTCAGTAAGACAAAAAGACCCTGTAGAGCTTCATTTTATCA

TTAACTCATAATTTTCATTCTATGTAATTTCCACAGATTAATGTAAAATTTAGTTGGGGCGACAAAGGACCACCCAAAAC

GTCCTTACACAACTAGACCATTTGTCACTAAACGACTCATTAGACACCTTGTTCAAGAAAAATTAGCTACCTCAGGGATA

ACAGGCTAATATCCCCTCGAGAGTTCACATTAACAGGGGAGCTTGGCACCTCGATGTTGGCTTAGGGAACCCAAACTAGT

GCAGCAGCTAGTTCAGGGCGGTTTGTTCAACCGATAACCCCCTACGTGATCTGA

>Raricirrus_anubis_16S

TCGCCTGTTTTATCAAAAACATTGCCTGTAGCCCACCCCATTATAGGTTCGTCCTGCCCAGTGAAATATTTAAACGGCCG

CGGTACCCTGACCGTGCTAAGGTAGCAAAATCAATTGCCCTTTAATTGGGGGCGCGTATGAATGGATCTACGAGAGCTAA

GCTGTCTCACACCTGCCTATGAAAACTAACCTTTAGGTGAAAAGGCCTAAATATACTAAAAAGACAAGAAGACCCTGTAG

AGCTTCACTCCCACAGTGTCGAACAGAGCTTATTTATAAATTTCTTGCACCACACCTAAAAAGAGTTTGGTTGGGGCAAC

CAGGGACTAACTAAACCGTCTCTTACCAACCTTTGACACATTTGTCTTACAAAAGACCCGTATATTAATATGACTCGAAG

AATTAGCTACCTAAGGGATAACAGACTAATCCCCCTTGAGAGTCCACATTGACAGGGGGGCTTGGCACCTCGATGTTGGC

TCAGGATACCCTTTATGTGCAGAAGCATTATAGGTAAGTTTGTTCACCTTTTAAAATCCTACGTGATCTGAGTTCAGACC

GG
